# Effects of Atorvastatin and Simvastatin on the Bioenergetic Function of Isolated Rat Brain Mitochondria

**DOI:** 10.3390/ijms25158494

**Published:** 2024-08-03

**Authors:** Krzysztof Wojcicki, Adrianna Budzinska, Wieslawa Jarmuszkiewicz

**Affiliations:** Mitochondrial Biochemistry Research Group, Faculty of Biology, Adam Mickiewicz University in Poznan, 61-614 Poznan, Poland; krzysztof.wojcicki@amu.edu.pl (K.W.); adrianna.budzinska@amu.edu.pl (A.B.)

**Keywords:** atorvastatin, simvastatin, brain mitochondria, respiration, membrane potential, reactive oxygen species

## Abstract

Little is known about the effects of statins, which are cholesterol-lowering drugs, on the bioenergetic functions of mitochondria in the brain. This study aimed to elucidate the direct effects of atorvastatin and simvastatin on the bioenergetics of isolated rat brain mitochondria by measuring the statin-induced changes in respiratory chain activity, ATP synthesis efficiency, and the production of reactive oxygen species (ROS). Our results in isolated brain mitochondria are the first to demonstrate that atorvastatin and simvastatin dose-dependently significantly inhibit the activity of the mitochondrial respiratory chain, resulting in a decreased respiratory rate, a decreased membrane potential, and increased ROS formation. Moreover, the tested statins reduced mitochondrial coupling parameters, the ADP/O ratio, the respiratory control ratio, and thus, the oxidative phosphorylation efficiency in brain mitochondria. Among the oxidative phosphorylation complexes, statin-induced mitochondrial impairment concerned complex I, complex III, and ATP synthase activity. The calcium-containing atorvastatin had a significantly more substantial effect on isolated brain mitochondria than simvastatin. The higher inhibitory effect of atorvastatin was dependent on calcium ions, which may lead to the disruption of calcium homeostasis in mitochondria. These findings suggest that while statins are effective in their primary role as cholesterol-lowering agents, their use may impair mitochondrial function, which may have consequences for brain health, particularly when mitochondrial energy efficiency is critical.

## 1. Introduction

The bioenergetic functioning of mitochondria is crucial for maintaining cellular energy homeostasis, especially in tissues with a high metabolic activity, such as the brain. Mitochondria are the powerhouses of the cell, producing ATP in the process of oxidative phosphorylation (OXPHOS), and are the main source of the formation of reactive oxygen species (ROS). Any disturbances in the bioenergetics of mitochondria may have serious consequences for cell functioning and viability. Several neurological disorders and chronic neurodegenerative diseases have their origins in mitochondrial dysfunction in the brain [1,2].

Atorvastatin and simvastatin, both members of the statin family, are widely used lipid-lowering drugs that inhibit 3-hydroxy-3-methylglutaryl coenzyme A (HMG-CoA) reductase, a key enzyme in the mevalonate pathway of cholesterol synthesis [3]. By inhibiting the synthesis of mevalonate, statins also reduce the synthesis of heme *a* (the cofactor of cytochrome *c* oxidase, complex IV, CIV) and coenzyme Q, which are essential components of the mitochondrial electron transport chain [4,5]. In addition to their cholesterol-lowering effects, statins affect mitochondrial function, potentially influencing cellular energy metabolism and redox balance, particularly in muscle cells.

Once statins penetrate the brain parenchyma, they exert pleiotropic effects on neuronal and glial cells, affecting neurotransmitter levels, neurotransmitter receptors, cell viability, neuronal dendrite arborization, and oligodendrocyte-mediated myelination [6,7]. Compared to hydrophilic statins, lipophilic statins such as atorvastatin and simvastatin, cross the blood–brain barrier more easily, which may lead to central nervous system complaints such as insomnia, confusion, anxiety, depression, dementia, and cognitive problems [7,8,9,10]. However, there is some interest in the use of statins in the treatment of certain neurodegenerative diseases (cerebrovascular diseases, Parkinson’s disease, Alzheimer’s disease, and multiple sclerosis) [6,7,9,10,11]. In this context, examining the effects of atorvastatin and simvastatin on the bioenergetic functioning of isolated rat brain mitochondria may provide valuable information on the broader implications of statin therapy on neurological health. The brain’s dependence on efficient mitochondrial function for energy production and the regulation of oxidative stress highlights the importance of understanding how these drugs may affect mitochondrial efficiency.

With this study, we aimed to explain the direct effects of atorvastatin and simvastatin on the bioenergetics of isolated brain mitochondria, emphasizing parameters such as respiratory chain activity, ATP synthesis efficiency and the production of mitochondrial ROS (mROS).

## 2. Results

Lipophilic statins can easily enter cells and concentrate in membrane compartments such as mitochondria. We investigated the effect of increasing concentrations of atorvastatin and simvastatin up to 200 µM on the oxidative metabolism of rat brain mitochondria.

### 2.1. In Rat Brain Mitochondria, Atorvastatin and Simvastatin Decrease Mitochondrial Respiratory Rates and Coupling Parameters

**Figure 1:**
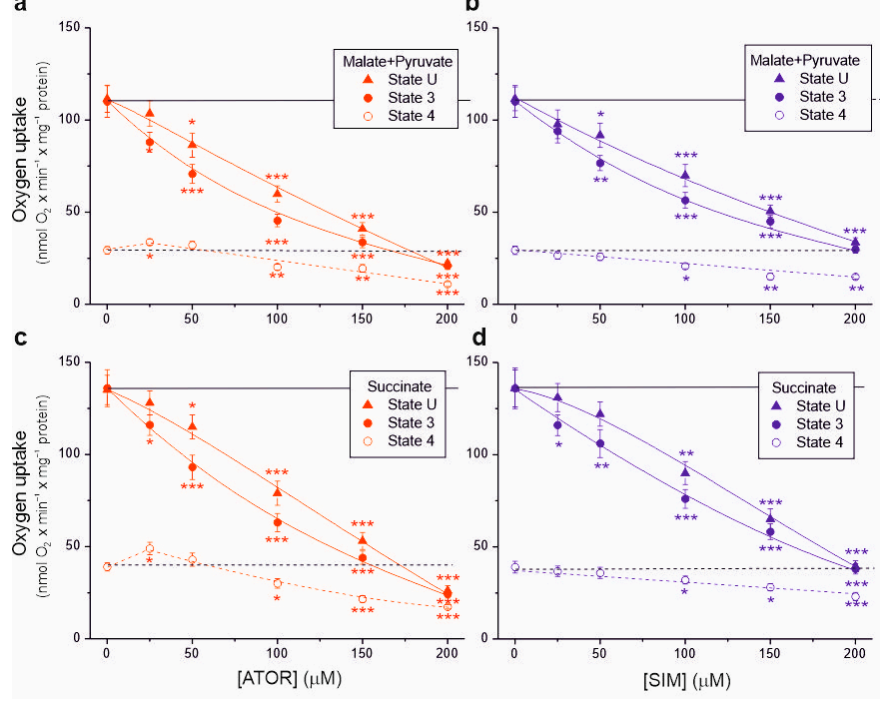
Effects of increasing concentrations of atorvastatin (ATOR) and simvastatin (SIM) on mitochondrial respiratory rates. Phosphorylating (State 3) respiration, uncoupled (State U) respiration, and non-phosphorylating (State 4) respiration with malate and pyruvate (**a**,**b**) or succinate (**c**,**d**) as respiratory substrates. Mean ± SD; *n* = 6; *p* < 0.05 (*), *p* < 0.01 (**), *p* < 0.001 (***), comparison vs. control conditions (horizontal lines).

In isolated brain mitochondria, we measured non-phosphorylating respiration and maximal respiratory chain activity (uncoupled and phosphorylating respiration) using complex I (malate and pyruvate) or complex II (succinate) substrates (Figure 1). A progressive decrease in respiratory rates (up to ~70–80%) was observed with increasing concentrations of atorvastatin and simvastatin in the uncoupled and phosphorylating mitochondria for these substrates. Generally, at a given concentration of atorvastatin or simvastatin, phosphorylating respiration was more inhibited than uncoupled respiration, indicating the inhibition of ATP synthesis in addition to the inhibition of the respiratory chain.

With 25 μM atorvastatin, but not simvastatin, the non-phosphorylating respiratory rate increased by 15% and 25% for complex I and complex II substrates, respectively, indicating the uncoupling of respiration (Figure 1a,c). At concentrations of both statins above 50 µM, the non-phosphorylating respiratory rate gradually decreased. At a concentration of 100 μM, the inhibitory effect induced by atorvastatin was more substantial than that induced by simvastatin under all respiratory conditions (Table 1). Furthermore, the inhibitory effect for both statins was more substantial with the malate–pyruvate mixture than with succinate, indicating a greater sensitivity of complex I substrate oxidation than complex II substrate oxidation.

**Figure 2:**
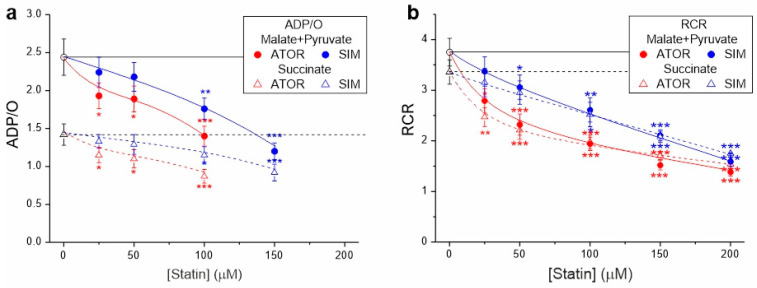
Effects of increasing concentrations of atorvastatin (ATOR) and simvastatin (SIM) on mitochondrial coupling parameters—ADP/O (**a**) and respiratory control ratio (RCR) (**b**)—with malate and pyruvate or succinate as respiratory substrates. RCR is the ratio of phosphorylating respiration to non-phosphorylating respiration. ADP/O ratio values for higher statin concentrations (above 100 µM for atorvastatin and above 150 µM for simvastatin) were not presented because their estimation with reduced OXPHOS coupling was imprecise and subject to large errors. Mean ± SD; *n* = 6; *p* < 0.05 (*), *p* < 0.01 (**), *p* < 0.001 (***), comparison vs. control conditions (horizontal lines).

In the case of both types of respiratory substrates, with increasing concentrations of both statins, a progressive decrease in ADP/O and the respiratory control ratio was observed, indicating the statin-induced impairment of OXPHOS (Figure 2). At a given concentration, the inhibitory effect of atorvastatin on mitochondrial coupling parameters was more substantial than that of simvastatin.

### 2.2. In Rat Brain Mitochondria, Statins Decrease Mitochondrial Membrane Potential under Non-Phosphorylating Conditions and Phosphorylating Conditions (at Concentrations above 50 µM)

**Figure 3:**
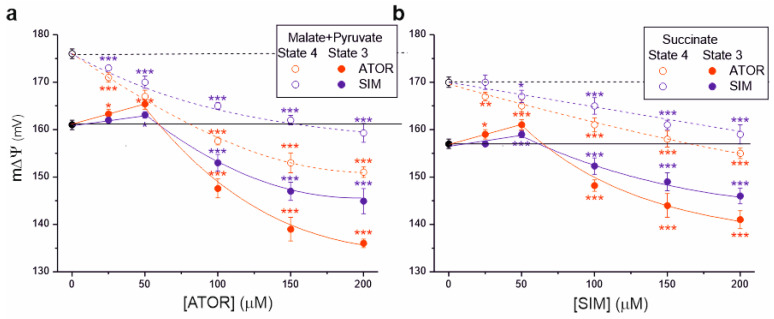
Effects of increasing concentrations of atorvastatin (ATOR) and simvastatin (SIM) on mitochondrial membrane potential (m∆Ψ). Non-phosphorylating (State 4) respiration, phosphorylating (State 3) respiration with malate and pyruvate (**a**) or succinate (**b**) as respiratory substrates. Mean ± SD; *n* = 5; *p* < 0.05 (*), *p* < 0.01 (**), *p* < 0.001 (***), comparison vs. control conditions (horizontal lines).

We then measured the effects of statins on another parameter, mitochondrial membrane potential (m∆Ψ), illustrating the polarization of inner mitochondrial membranes (Figure 3). For both complex I and complex II respiratory substrates, with increasing statin concentrations, a progressive decrease in m∆Ψ was observed under non-phosphorylating conditions and phosphorylating conditions (at concentrations above 50 µM), indicating a progressive inhibition of OXPHOS. For a given statin concentration (above 50 µM), the inhibitory effect of atorvastatin on m∆Ψ was stronger than that of simvastatin in both respiratory states. The maximal statin-induced decrease in m∆Ψ was greater for malate and pyruvate oxidation (Figure 3a) than for succinate oxidation (Figure 3b).

Interestingly, under phosphorylating conditions at lower statin concentrations (up to 50 µM), an increase in m∆Ψ was observed, followed by a gradual inhibition (above 50 µM) (Figure 3). This effect was not observed under non-phosphorylating conditions for complex I and complex II respiratory substrates. These observations indicate that statins first inhibit ATP synthase activity (with an accompanying increase in m∆Ψ) and then the activity of the respiratory chain (with an accompanying decrease in m∆Ψ).

### 2.3. In Rat Brain Mitochondria, Statins Increase H_2_O_2_ Formation under Non-Phosphorylating and Phosphorylating Conditions

**Figure 4:**
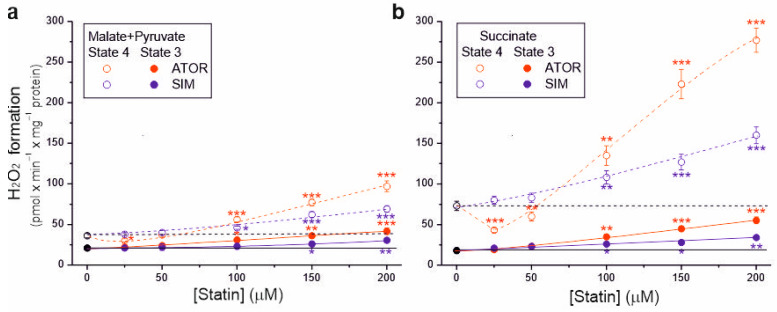
Effects of increasing concentrations of atorvastatin (ATOR) and simvastatin (SIM) on mitochondrial H_2_O_2_ formation. Non-phosphorylating (State 4) respiration and phosphorylating (State 3) respiration, with malate and pyruvate (**a**) or succinate (**b**) as respiratory substrates. Mean ± SD; *n* = 5; *p* < 0.05 (*), *p* < 0.01 (**), *p* < 0.001 (***), comparison vs. control conditions (horizontal lines).

Because the tested statins at higher concentrations (above 50 µM) reduced respiratory rates, m∆Ψ, and coupling parameters, their effects on mROS production in the resting (non-phosphorylating) and maximal (phosphorylating) respiratory states was also examined (Figure 4). Without statin, during malate and pyruvate oxidation, H_2_O_2_ formation was ~21 and ~36 nmol H_2_O_2_ × min × mg^−1^ protein under phosphorylating and non-phosphorylating conditions, respectively (Figure 4a). However, during succinate oxidation, H_2_O_2_ formation was ~18 and ~73 nmol H_2_O_2_ × min × mg^−1^ protein under phosphorylating and non-phosphorylating conditions, respectively (Figure 4b). Therefore, a lower membrane potential in the phosphorylating state resulted in a lower mROS production, and a higher membrane potential in the non-phosphorylating state resulted in a higher mROS production (Figure 3 and Figure 4).

During both the oxidation of complex I and complex II respiratory substrates, with increasing statin concentration (above 50 µM), a gradual increase in H_2_O_2_ production was observed, which was higher in the non-phosphorylating state than in the phosphorylating state. Compared to control conditions, the highest concentration of atorvastatin (200 µM) increased H_2_O_2_ production ~2-fold and ~3.1-fold under phosphorylating conditions for malate plus pyruvate and succinate, respectively, and ~2.7-fold and ~3.8-fold under non-phosphorylating conditions for malate plus pyruvate and succinate, respectively. The same concentration of simvastatin increased H_2_O_2_ production ~1.4-fold and ~1.9-fold under phosphorylating conditions for malate plus pyruvate and succinate, respectively, and ~1.9-fold and ~2.2-fold under non-phosphorylating conditions for malate plus pyruvate and succinate, respectively. Therefore, for both types of substrates and both respiratory states, at a given statin concentration (above 50 µM), the effect of atorvastatin on H_2_O_2_ production was more substantial compared to simvastatin. Moreover, the maximum statin-induced increase in H_2_O_2_ production was higher for succinate oxidation (Figure 4b) than for malate and pyruvate oxidation (Figure 4a).

For 25 µM atorvastatin, but not simvastatin, H_2_O_2_ production under non-phosphorylating conditions decreased by 17% and 40% for complex I and complex II substrates, respectively, indicating the uncoupling of respiration (Figure 4). With higher atorvastatin concentrations, H_2_O_2_ production gradually increased, indicating that the observed uncoupling effect was transient. Under non-phosphorylating conditions, the transient reduction in mROS production induced by atorvastatin (Figure 4), accompanied by a transient increase in respiration (Figure 1a,c), is likely attributable to the uptake of Ca^2+^ contained in atorvastatin.

### 2.4. Atorvastatin and Simvastatin Inhibit the Activity of Complex I, Complex III, and ATP Synthase of Rat Brain Mitochondria

**Figure 5:**
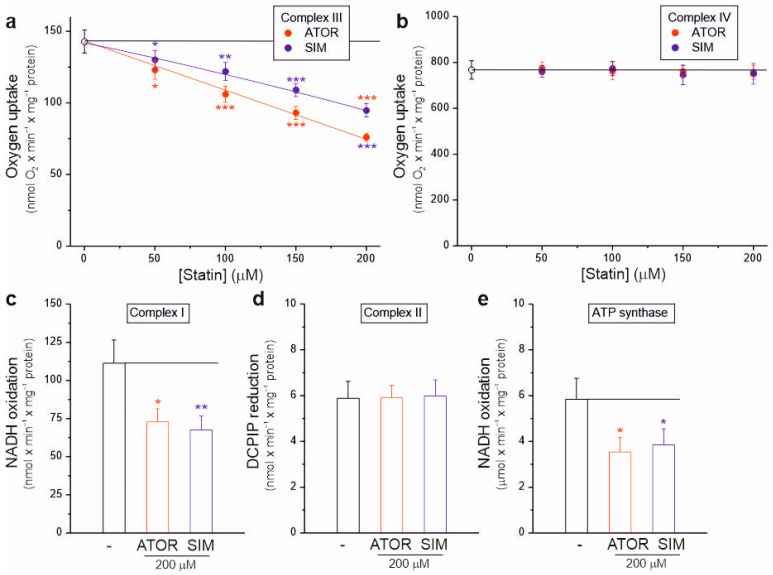
Effects of atorvastatin (ATOR) and simvastatin (SIM) on individual OXPHOS complexes. Complex III (**a**) and complex IV (**b**) activity with increasing concentrations of statin in isolated intact rat brain mitochondria. Activity of complex I (**c**), complex II (**d**), and ATP synthase (**e**) in the absence or presence of 200 µM statins in rat brain mitochondrial lysate. Mean ± SD; *n* = 4; *p* < 0.05 (*), *p* < 0.01 (**), *p* < 0.001 (***), comparison vs. control conditions (horizontal line).

Our results in isolated brain mitochondria show that atorvastatin and simvastatin dose-dependently significantly inhibit the activity of the mitochondrial respiratory chain, resulting in decreased respiratory rates (Figure 1), decreased mΔΨ (Figure 3), and increased mROS formation (Figure 4). Therefore, we next examined the sensitivity of individual OXPHOS complexes to statins. Using intact rat brain mitochondria, we examined the maximal activity of complex III and complex IV in the absence or presence of increasing concentrations of statins (Figure 5a,b). To test whether complex III activity is sensitive to statins, we used duroquinol, a direct electron donor for coenzyme Q that bypasses dehydrogenases, as a respiratory substrate (Figure 5a). At maximal respiratory chain activity under uncoupling conditions, duroquinol oxidation was gradually inhibited up to 46% and 32% by increasing the concentrations of atorvastatin and simvastatin, respectively. These results indicate that both statins inhibit complex III activity, with atorvastatin being the more potent inhibitor. However, complex IV activity did not change with increasing statin concentrations (Figure 5b).

We also examined the activity of dehydrogenases (complex I and complex II) and ATP synthase in the absence or presence of 200 µM atorvastatin or simvastatin in the rat brain mitochondrial lysate with disrupted membranes (Figure 5c–e). Enzymatic activity measurements indicate that both statins similarly inhibited the activity of complex I (Figure 5c) and ATP synthase (Figure 5e) by ~35–40%, while the activity of complex II remained unchanged (Figure 5d).

Therefore, our results showed that atorvastatin and simvastatin could inhibit the activities of complex I, complex III, and ATP synthase (Figure 5), confirming previous studies on the functioning of the rat brain mitochondrial respiratory chain and OXPHOS under different bioenergetic conditions (Figure 1 and Figure 2).

### 2.5. The Effect of Atorvastatin on the Bioenergetic Functioning of Mitochondria Is Ca^2+^-Dependent

**Figure 6:**
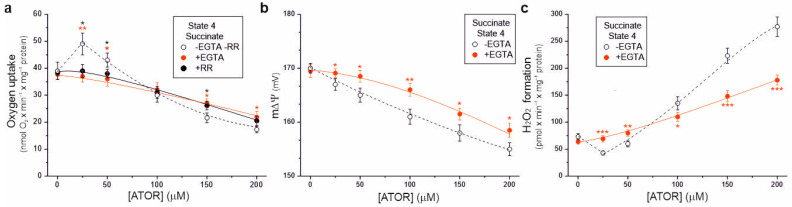
Ca^2+^-dependent effects of increasing concentrations of atorvastatin (ATOR) on mitochondrial respiratory rate (**a**), membrane potential (m∆Ψ) (**b**), and H_2_O_2_ formation (**c**) under non-phosphorylating conditions (State 4) with succinate as the respiratory substrate. Measurements were performed in the absence or presence of 0.4 mM EGTA (**a**–**c**) or 2 µM ruthenium red (RR) (**a**). Mean ± SD; *n* = 5; *p* < 0.05 (*), *p* < 0.01 (**), *p* < 0.001 (***), comparison vs. values obtained without EGTA and RR.

To determine whether the effects of atorvastatin on the bioenergetic functioning of isolated rat brain mitochondria depend on the calcium ions contained in this hydrophobic molecule, we used the Ca^2+^ chelator ethylene glycol-bis(β-aminoethyl)-*N*,*N*,*N*′,*N*′-tetraacetic acid (EGTA) and the Ca^2+^ uptake inhibitor ruthenium red (Figure 6). In the presence of EGTA, for atorvastatin concentrations up to 50 μM, during succinate oxidation under non-phosphorylating conditions, no transient increase in respiratory rate (Figure 6a) or decrease in H_2_O_2_ formation (Figure 6c) was observed compared to measurements without EGTA, indicating that these transient changes were caused by uncoupling due to the entry of Ca^2+^. This observation was confirmed by the lack of transient stimulation of respiratory rate in the presence of ruthenium red (Figure 6a). In the presence of EGTA, at higher atorvastatin concentrations (above 50 µM), a progressive reduction in the respiratory rate, m∆Ψ, and increased H_2_O_2_ formation were observed (Figure 6). However, these effects were weaker than those obtained in the absence of EGTA. Thus, our findings indicate that under non-phosphorylating conditions, atorvastatin caused Ca^2+^-dependent mitochondrial uncoupling and the subsequent (at higher concentrations) inhibition of respiration, accompanied by an increase in mROS production.

## 3. Discussion

To investigate whether lipophilic statins could directly affect brain mitochondria, we first performed acute in vitro experiments with isolated rat brain mitochondria treated with atorvastatin or simvastatin. Both statins tested did not alter brain mitochondrial oxidative capacities, i.e., complex IV activity (Figure 5b) and citrate synthase activity (Supplementary Figure S1a), although 200 μM atorvastatin slightly reduced outer mitochondrial membrane integrity (Supplementary Figure S1b). However, atorvastatin and simvastatin had a substantial effect on brain mitochondria’s functional properties, probably due to their concentration in mitochondrial membranes. The more substantial effect of atorvastatin compared to simvastatin (at a given concentration) (Table 1) may be related to the presence of calcium ions in the atorvastatin molecule, the accumulation of which by the calcium uniporter in the inner mitochondrial membrane is driven by m∆Ψ [12]. The concentrations of both drugs in the blood of statin-treated patients (up to 0.2 µM, [13,14]) were significantly higher than the concentrations of atorvastatin and simvastatin (up to 200 µM) applied to isolated rat brain mitochondria in this study. However, intracellular, cytosolic, and mitochondrial concentrations of statins in the brain and other cells are unknown. The potential accumulation of lipophilic drugs in mitochondria in vivo, particularly in hydrophobic mitochondrial membranes [15], justifies using higher concentrations of lipophilic statins in acute in vitro studies with isolated mitochondria. This approach may help us to understand the direct effects of statins on mitochondria and the potential toxicity of these drugs, which may only become apparent at higher, localized concentrations.

The effect of statins on brain mitochondria has not been studied so far. Our results in isolated brain mitochondria are the first to demonstrate that atorvastatin and simvastatin dose-dependently significantly inhibit the activity of the mitochondrial respiratory chain, resulting in decreased respiratory rates (Figure 1), decreased m∆Ψ (Figure 3), and increased mROS formation (Figure 4). Because the tested statins did not affect the activity of complex IV and complex II, but significantly inhibited the activity of complex I and complex III (Figure 5), and had a more substantial adverse effect on complex I substrate oxidation than complex II substrate oxidation (Table 1), it can be concluded that the statin-induced impairment of the mitochondrial respiratory chain mainly affects complex I and complex III. These results therefore indicate that the inhibition of the oxidation of complex I substrates (malate and pyruvate) by statins may result mainly from the inhibition of the respiratory chain (complexes I and III). The weaker inhibition of complex II substrate (succinate) oxidation induced by statins may be mainly due to the inhibition of the respiratory chain at the level of complex II. However, the potential inhibition of succinate transport into mitochondria by statins cannot be excluded. In rat brain mitochondria, atorvastatin and simvastatin reduced mitochondrial coupling parameters, the ADP/O ratio, and the respiratory control ratio (Figure 2). Moreover, at a given concentration, they inhibited phosphorylating respiration more than they inhibited uncoupled respiration (Figure 1), indicating the statin-induced impairment of OXPHOS in addition to the inhibition of the respiratory chain. Enzymatic measurements showed the inhibition of ATP synthase activity by the statins tested (Figure 5e). All observed statin-induced effects in isolated rat brain mitochondria were not related to the inhibition of the mevalonate pathway, which occurs in the cytosol, but to a direct effect on mitochondrial function. Therefore, brain mitochondrial dysfunction induced by statins administered in vitro (this study) was not associated with coenzyme Q deficiency, reduced protein prenylation, or the induction of the mitochondrial apoptosis pathway, which are mechanisms of statin action proposed based on in vivo studies with cultured cells [4,5].

Our results are consistent with the few previous studies that have reported statin-induced mitochondrial dysfunction, manifested by respiratory chain impairment, increased mROS formation, and mΔΨ depolarization, which have been previously observed in mitochondria isolated from pancreatic cells, muscle cells, and endothelial cells [16,17,18,19]. Namely, in isolated pancreatic mitochondria, complex II activity was inhibited by atorvastatin but not by simvastatin [17]. In isolated skeletal muscle mitochondria, both statins inhibited the oxidation of the complex I substrate (CI + CIII + CIV pathway), and the inhibitory effect of atorvastatin was much stronger than that of simvastatin [16]. Similar to isolated brain mitochondria (in this study), in isolated endothelial mitochondria, atorvastatin reduced the activity of the CI + CIII + CIV pathway (malate oxidation) and CII + CIII + CIV pathway (succinate pathway) more effectively than simvastatin [18]. Moreover, in this study, a significant inhibition of duroquinol-driven complex III activity was observed in rat brain mitochondria (Figure 5a). However, simvastatin lactone, but not acid, have been shown to inhibit the Q_o_ site of complex III in isolated bovine heart mitochondria [19]. Thus, observations from in vitro studies indicate that all complexes of the mitochondrial respiratory chain (except complex IV) may be susceptible to the inhibitory effects of both direct-acting statins (especially atorvastatin). Interestingly, complex III activity was reduced in the muscle biopsies of patients with statin-induced myopathy, which is the most common side effect of these commonly used cholesterol-lowering drugs [19]. Moreover, the reduced content of coenzyme Q10 in the muscles of statin-treated patients may impair mitochondrial function [20,21].

Studies of isolated endothelial cell mitochondria [18] and brain mitochondria (Figure 1 and Figure 2) and enzymatic measurements (Figure 5e) indicate that statins can inhibit ATP synthase activity in vitro, leading to impaired OXPHOS efficiency. Interestingly, to our knowledge, no studies have been performed on ATP synthase activity in mitochondria isolated from cells or animals treated with statins. Therefore, assessing whether the reduced ATP level in cells or animals treated with statins [4,5,21] results only from inhibiting the mitochondrial respiratory chain or also ATP synthase is difficult.

Studies on statin-induced mitochondrial dysfunction in animal and human models have shown increased Ca^2+^ efflux from the sarcoplasmic reticulum, leading to abnormalities in excitation–contraction coupling in skeletal muscle cells [22]. However, the effect of statins on mitochondrial calcium homeostasis has not been thoroughly investigated. In our study on isolated brain mitochondria, under non-phosphorylating conditions, calcium-containing atorvastatin (25 µM), but not simvastatin, caused mitochondrial uncoupling, manifested by an increased respiratory rate, a reduced mΔΨ, and mROS formation, as well as the subsequent inhibition of respiration at higher statin concentrations (Figure 1, Figure 3 and Figure 4). Therefore, under non-phosphorylating conditions, atorvastatin caused mitochondrial uncoupling followed by the inhibition of respiration. Measurements in the presence or absence of the Ca^2+^ chelator EGTA indicate that atorvastatin’s effect on the bioenergetic functions of brain mitochondria is primarily Ca^2+^ dependent (Figure 6). Measurements of respiratory rate in the presence or absence of the mitochondrial calcium uniporter (ruthenium red) (Figure 6a) indicate that the direct effects of a Ca^2+^-containing statins on mitochondria are related to Ca^2+^ uptake. Similar findings that the direct effects of Ca^2+^-containing atorvastatin on mitochondria are linked to Ca^2+^ uptake, which may alter mitochondrial Ca^2+^ homeostasis and lead to mitochondrial swelling, have been found in isolated endothelial mitochondria [18]. Our results indicate that calcium ions may enhance atorvastatin-induced mitochondrial impairment. Moreover, unlike calcium-free simvastatin, the highest concentration of atorvastatin (200 μM) led to a slight loss of outer mitochondrial membrane integrity (Supplementary Figure S1b), indicating that calcium ions may also compromise mitochondrial integrity. It should be remembered that in the case of atorvastatin, it is difficult to dissect which effects described (Figure 1, Figure 2, Figure 3, Figure 4 and Figure 5) resulted mainly from the effect of the statin itself and not the addition of calcium ions. Other Ca^2+^-containing statins, pitavastatin or rosuvastatin, have not yet been studied in this context. However, it has previously been observed both in vivo and in vitro in animal and human models that statins, which do not necessarily contain Ca^2+^ (e.g., simvastatin), increase cytosolic and sarcoplasmic Ca^2+^ levels likely through mitochondrial Ca^2+^ efflux mediated by the mitochondrial permeability transition pore and the Na+/Ca^2+^ exchanger in myocytes [23]. The effect of statins on mitochondrial calcium homeostasis in other cells requires further study.

In vitro studies on isolated mitochondria from pancreatic [17], endothelial [18], and brain (this study) cells, as well as in vivo and in vitro studies in animal and human models [4,5], indicate that the statin-induced inhibition of the mitochondrial respiratory chain may lead to the increased production of mROS and thus oxidative stress. Excessive oxidative stress can impair mitochondrial proteins, lipids, and DNA, leading to mitochondrial dysfunction and cell death. This is particularly dangerous for brain cells, which are highly susceptible to oxidative damage due to their high metabolic rates and limited regenerative capacity. The brain relies heavily on the efficient functions of mitochondria for energy production. Increased oxidative stress induced by statins may impair neuronal function and viability, potentially contributing to cognitive decline and neurodegenerative diseases. Surprisingly, little is known about the effects of statins on mitochondrial function in the brain. However, many studies have been published on the relationship between statin treatment and cognitive functions [5,10]. These studies often provide conflicting results, demonstrating statins’ protective or harmful effects on cognitive function. How the changes in mitochondrial function explain these contradictory results has not been elucidated. Although our in vitro results on isolated brain mitochondria cannot be simply transferred to in vivo conditions with the more complex regulation of mitochondrial oxidative metabolism, they provide potential statin-sensitive sites related to mitochondrial bioenergetic activity.

## 4. Materials and Methods

### 4.1. Chemicals

All chemicals used in the experiments, including atorvastatin (1044516) and simvastatin (1612700), were purchased from Sigma-Aldrich (St. Louis, MO, USA). Atorvastatin was dissolved in methanol. Before use in in vitro studies, simvastatin must be activated by opening the lactone ring. To prepare a 100 mM simvastatin solution, 16.7 mg of simvastatin was dissolved in 0.2 mL of methanol, and then 0.1 mL of 100 mM NaOH was added. The solution was heated at 60 °C for ~2 h and was then neutralized with HCl to pH 7.2. The resulting solution was adjusted to the final volume (0.4 mL) with distilled water, and aliquots were stored at −20 °C until use. Control measurements (without statins) were performed with a given amount of appropriate solvent.

### 4.2. Animals

The experiments were carried out on 3–4-month-old male Wistar rats weighing 350–450 g. The animals had free access to water and food and were maintained under standard temperature and humidity conditions, with a 12 h light/dark cycle. The protocols for animal experiments, surgery, and care followed the European Community Council Directive guidelines on the protection of animals used for scientific purposes. The animals were euthanized by decapitation, and every effort was made to minimize suffering. Because no procedures were performed on live animals, no approval was required for our study following the Polish Animal Welfare Act.

### 4.3. Mitochondria Isolation

All procedures for isolating mitochondria from freshly collected brains were performed at 4 °C. Brains placed in isolation medium A (pH 7.2) containing 10 mM Tris-HCl, 100 mM sucrose, and 0.5 mM ethylenediaminetetraacetic acid (EDTA) were washed several times. Brains were cut into small pieces on ice. The minced tissue was homogenized using a Dounce homogenizer in isolation medium B (pH 7.4) containing 10 mM Tris-HCl, 300 mM sucrose, 0.5 mM EDTA, and 0.2% bovine serum albumin (BSA). The homogenates were centrifuged at 2000× *g* for 5 min. The homogenate pellets were suspended in isolation medium C (pH 7.2) containing 10 mM Tris-HCl, 300 mM sucrose, and 0.5 mM EDTA, and were centrifuged again. Supernatants from both centrifugations were centrifuged at 12,000× *g* for 15 min. The obtained pellets of crude mitochondria were suspended in isolation medium C and were centrifuged in a self-generating Percoll gradient (14% Percoll, 10 mM Tris-HCl (pH 7.2), 300 mM sucrose, and 0.5 mM EDTA) at 40,000× *g* for 40 min. The collected fractions of purified mitochondria were suspended in isolation medium C and were centrifuged at 12,000× *g* for 15 min. After washing again, the final mitochondrial pellets were resuspended in isolation medium C. Mitochondrial protein concentrations were determined using the Bradford method. To measure the enzymatic activities of complex I, complex II, and ATP synthase, mitochondria were stored at −80 °C.

### 4.4. Measurements of Mitochondrial Respiration and Membrane Potential

Oxygen consumption was determined polarographically using a Rank Bros. (Cambridge, UK) oxygen electrode or a Hansatech oxygen electrode with either 1.5 or 0.3 mg of mitochondrial protein in either 3.0 or 0.6 mL of standard incubation medium (at 34 °C), respectively, which consisted of 75 mM sucrose, 225 mM mannitol, 5 mM KCl, 5 mM KH_2_PO_4_, 0.5 mM EDTA, 10 mM Tris/HCl (pH 7.2), and 0.1% BSA. The membrane potential was measured simultaneously with oxygen uptake using a tetraphenylphosphonium (TPP^+^)-specific electrode, as previously described [24]. The TPP^+^ electrode was calibrated by three successive additions (0.8, 0.8, and 1.6 μM) of TPP^+^. After each run, up to 1 µM FCCP was added to release TPP^+^ for baseline correction. To calculate the membrane potential value, the volume of the brain mitochondrial matrix was assumed to be 2.0 µL × mg^−1^ protein. The calculations assumed that the distribution of TPP^+^ between mitochondria and the medium follows the Nernst equation.

Respiratory substrates of complex I (5 mM malate and 5 mM pyruvate) and complex II (5 mM succinate plus 0.2 μM rotenone) were used. Phosphorylating respiration (state 3) was measured in the presence of 150 μM (pulse) or 1 mM (saturating) ADP. The efficiency of OXPHOS was assessed by calculating the coupling parameters, i.e., respiratory control ratio (RCR) and ADP/O ratio. The ADP/O ratio was determined using an ADP pulse method. The total amount of oxygen consumed during phosphorylating respiration was used to calculate the ratio. The simultaneous measurements of membrane potential enabled the fine control of the duration of state 3. RCR was calculated as the ratio of phosphorylating respiration to non-phosphorylating respiration. Uncoupled respiration (state U) was measured in the presence of up to 1 μM carbonyl cyanide-*p*-trifluoromethoxyphenylhydrazone (FCCP). Non-phosphorylating (state 4, resting state) respiration was measured after exogenous ADP depletion.

### 4.5. Measurements of the Activity of Mitochondrial Respiratory Chain Complexes and ATP Synthase Activity

Mitochondrial enzyme activity measurements were performed at 34 °C under constant stirring.

The maximal activity of complex III (ubiquinol–cytochrome *c* oxidoreductase) was assessed by measuring oxygen consumption of isolated mitochondria under uncoupling conditions (in the presence of 1 μM FCCP) with 1 mM duroquinol as the respiratory substrate. The activity was assessed in 0.6 mL of standard incubation medium (Section 4.4) with 0.3 mg of mitochondrial protein.

The maximal complex IV (cytochrome *c* oxidase) activity was assessed by measuring the oxygen consumption of isolated mitochondria, as previously described [25]. The activity was assessed in 0.6 mL of standard incubation medium (Section 4.4) with 60 µg of mitochondrial protein without respiratory substrates and in the presence of sequentially added antimycin A (1 µg/mL), 10 mM ascorbate, 0.05% cytochrome *c*, and up to 1 mM *N*,*N*,*N*′*N*′-tetramethyl-*p*-phenylenediamine (TMPD). The rate of oxygen consumption following the addition of TMPD reflected the maximal O_2_ consumption of complex IV.

Complexes I and II and ATP synthase activities were measured in mitochondrial lysate, as previously described [26] with modifications. Frozen isolated mitochondria were thawed and pelleted by centrifuging the samples at 12,000× *g* for 10 min at 4 °C. The mitochondrial pellet was resuspended in hypotonic buffer (10 mM Tris/HCl pH 7.6) with a final protein concentration of 1 µg/µL. Four freeze–thaw cycles were then performed to increase mitochondrial membrane disruption. The enzyme activities of complex I, complex II, and ATP synthase were determined spectrophotometrically using a Shimadzu 1620 UV spectrophotometer. The activity of complex I (NADH:ubiquinone oxidoreductase) was measured by monitoring NADH oxidation at 340 nm in 0.8 mL of a reaction mixture containing 50 mM potassium buffer, pH 7.5; 0.3% BSA; 0.5 mM cyanide; 2 µg/mL antimycin A; 120 µM NADH; 100 µM duroquinone; and 30 µg of mitochondrial lysate in the absence and presence of 20 µM rotenone (complex I inhibitor) [26]. The activity of complex II (succinate:ubiquinone oxidoreductase) was measured by monitoring dichloroindophenol (DCPIP) reduction at 600 nm in 0.8 mL of a reaction mixture containing 25 mM potassium buffer, pH 7.5; 0.1% BSA; 0.5 mM cyanide; 2 µg/mL antimycin A; 10 mM succinate; 75 µM DCPIP; 100 µM duroquinone; and 15 µg of mitochondrial lysate in the absence and presence of 20 mM malonate (complex II inhibitor) [26]. ATP synthase activity was measured by monitoring NADH oxidation in coupled enzyme reactions at 340 nm in 0.8 mL of a reaction mixture containing 50 mM HEPES buffer, pH 8.0; 5 mM MgCl_2_; 0.5 mM cyanide; 2 µg/mL antimycin A; 120 µM NADH; 50 µg/mL pyruvate kinase; 20 U lactate dehydrogenase; 4 mM phosphoenolpyruvate; 5 mM ATP; and 1.5 µg of mitochondrial lysate in the absence and presence of 10 µM oligomycin (ATP synthase inhibitor) [26]. The molar extinction coefficients of NADH (complex I and ATP synthase) and DCPIP (complex II) were used to calculate enzyme activities. Non-specific activity measured in the presence of the appropriate inhibitor was taken into account.

### 4.6. Measurements of Mitochondrial H_2_O_2_ Formation

Mitochondrial H_2_O_2_ production was measured using an Amplex red assay. The fluorescence kinetics of resorufin formation were followed for 40 min at an excitation wavelength of 545 nm and an emission wavelength of 590 nm using a Tecan Infinite M200 PRO plate reader [27]. Mitochondria (0.15 mg of mitochondrial protein) were incubated in 0.5 mL of the standard incubation medium (Section 4.4) with 0.1 U/mL horseradish peroxidase, 1 U/mL superoxide dismutase, and 5 μM Amplex red reagent. Measurements were performed with complex I (5 mM malate and 5 mM pyruvate) or complex II (5 mM succinate) respiratory substrates. For measurements in the phosphorylating state, 1.2 mM ADP was added. H_2_O_2_ production under non-phosphorylating conditions was measured after exogenous ADP depletion. The rate of H_2_O_2_ production was quantified with known amounts of H_2_O_2_.

### 4.7. Statistical Analysis

Data are presented as means ± SD obtained from at least 4 to 6 independent mitochondrial isolations. Each determination was performed at least three times. One-way ANOVA followed by a post hoc Tukey test for pairwise comparisons was used to identify significant differences. Differences between statin-treated and control mitochondria were statistically significant when *p* < 0.05 (*), *p* < 0.01 (**), and *p* < 0.001 (***).

## 5. Conclusions

Our studies have shown that the lipophilic statins, atorvastatin and simvastatin, which are widely used as anti-atherosclerotic drugs, have a direct adverse effect on isolated brain mitochondria, leading to the impairment of respiratory chain activity and OXPHOS efficiency, as well as an increased mROS formation. Among the OXPHOS complexes, statin-induced impairment affects complex I, complex III, and ATP synthase. The stronger effect of atorvastatin compared to simvastatin is related to the presence of calcium ions in the atorvastatin molecule and may lead to disturbances in mitochondrial calcium homeostasis. The brain is highly susceptible to oxidative damage and is dependent on the energy provided by mitochondrial metabolism. Therefore, our in vitro results suggest that the statin-induced adverse events observed in some statin-treated patients may be due to a direct effect of hydrophobic statins, which readily concentrate in mitochondria, on the bioenergetic functions of these organelles in the brain. The question remains whether statins can cause brain dysfunction by impairing brain mitochondrial function in vivo.

## Figures and Tables

**Table 1:** Comparison of the inhibitory effects (percentage inhibition) of 100 µM atorvastatin and 100 µM simvastatin on respiratory rates in isolated rat brain mitochondria.

	Malate + Pyruvate			Succinate		
	State 4	State 3	State U	State 4	State 3	State U
Inhibition (%) by 100 µM ATOR	31.1 ± 2.3 ###	59.6 ± 3.8 ** #	46.3 ± 3.3 ** #	21.1 ± 1.7 *	52.6 ± 3.9 **	41.9 ± 3.1 **
Inhibition (%) by 100 µM SIM	28.6 ± 2.0 ###	48.6 ± 2.8 #	37.3 ± 2.9 #	17.9 ± 1.6	43.1 ± 3.1	32.8 ± 2.8

State 4, non-phosphorylating respiration; State 3, phosphorylating respiration in the presence of ADP; State U, uncoupled respiration in the presence of 1 µM FCCP. Respiratory substrates: succinate or malate plus pyruvate. Mean ± SD; *n* = 6. *p* < 0.05 (*, #), *p* < 0.01 (**), *p* < 0.001 (###); *, **, comparison of atorvastatin-induced inhibition vs. simvastatin-induced inhibition; #, ###, comparison of statin-induced inhibition with malate–pyruvate vs. statin-induced inhibition with succinate for a given statin.

## Data Availability

The data that support the findings of this study are available on request from the corresponding author (W.J.).

## Supplementary Materials

The following supporting information can be downloaded at: https://www.mdpi.com/article/10.3390/ijms25158494/s1.
