# Supplementary material for: Effects of Atorvastatin and Simvastatin on the Bioenergetic Function of Isolated Rat Brain Mitochondria
Source: Int J Mol Sci. 2024 Aug 3;25(15):8494. doi: 10.3390/ijms25158494 (PMC11313418; doi:10.3390/ijms25158494)
Supplement: Supplementary file 1 [file ijms-25-08494-s001.zip › ijms-3111142-supplementary.pdf]

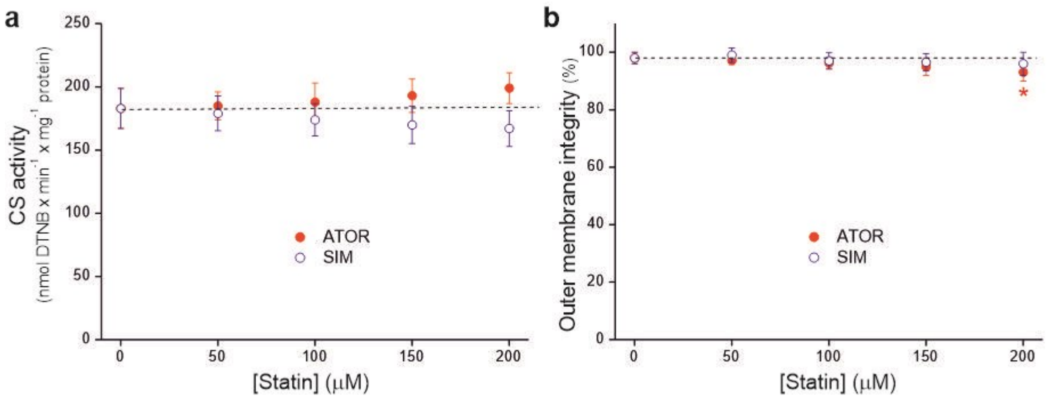

Figure S1. Dose-dependent effects of atorvastatin (ATOR) and simvastatin (SIM) on citrate synthase (CS) activity (**a**) and outer mitochondrial membrane integrity (**b**). Mean ± SD; n = 6; P < 0.05 (*), comparison vs. control conditions (horizontal lines).

Citrate synthase activity was measured spectrophotometrically by monitoring the formation of 5,5'-di-thiobis-(2-nitrobenzoic acid)-coenzyme A (DTNB-CoA) at 412 nm as previously described [25]. The reaction medium contained 100 mM Tris/HCl (pH 8.0), 100 µM DTNB), 100 µM acetyl CoA, 0.1% Triton X-100, and 100 µM oxaloacetate, with 60 µg of mitochondrial protein. Both statins used did not change the maximal activity of citrate synthase (Figure S1a).

The integrity of the outer mitochondrial membrane was assessed by comparing cytochrome *c* oxidase (complex IV) activity during measurements of oxygen uptake by isolated mitochondria in the presence and absence of exogenous cytochrome as previously described [25]. The activity was assessed in 0.6 mL of standard incubation medium (Section 4.4) with 60 µg of mitochondrial protein without respiratory substrates and in the presence of sequentially added antimycin A (1 µg/mL), 10 mM ascorbate, 0.05% cytochrome *c*, and up to 1 mM *N*,*N*,*N*'*N*'-tetramethyl-*p*-phenylenediamine (TMPD). No or slight increase in respiration after the addition of exogenous cytochrome *c* before the addition of TMPD indicated high outer membrane integrity.The integrity was calculated from oxygen consumption rates (OCR) in the presence of given chemicals using the following equation: $[TMPD_{OCR} – cytochrome\ c_{OCR}] / [TMPD_{OCR} – ascorbate_{OCR}] \times 100\%$. The procedure used to isolate rat brain mitochondria did not significantly reduce the integrity of the outer mitochondrial membrane, which was 98 ± 2% (Figure S1b). Unlike simvastatin, the highest concentration of atorvastatin (200 µM) led to a slight loss of outer mitochondrial membrane integrity from 98 ± 2% (control) to 93 ± 3%.
